# Dividing the Topological Charge of a Laguerre–Gaussian Beam by 2 Using an Off-Axis Gaussian Beam

**DOI:** 10.3390/mi13101709

**Published:** 2022-10-11

**Authors:** Alexey A. Kovalev, Victor V. Kotlyar, Elena S. Kozlova, Muhammad Ali Butt

**Affiliations:** 1 Laser Measurements Laboratory, Image Processing Systems Institute of the RAS—Branch of FSRC “Crystallography and Photonics” of the RAS, 151 Molodogvardeyskaya St., Samara 443001, Russia; 2 Technical Cybernetics Department, Samara National Research University, 34 Moskovskoe Shosse, Samara 443086, Russia; 3 Institute of Microelectronics and Optoelectronics, Warsaw University of Technology, Koszykowa 75, 00-662 Warszawa, Poland

**Keywords:** optical vortex, topological charge, Laguerre–Gaussian beam, off-axis Gaussian beam

## Abstract

In optical computing machines, many parameters of light beams can be used as data carriers. If the data are carried by optical vortices, the information can be encoded by the vortex topological charge (TC). Thus, some optical mechanisms are needed for performing typical arithmetic operations with topological charges. Here, we investigate the superposition of a single-ringed (zero-radial-index) Laguerre–Gaussian (LG) beam with an off-axis Gaussian beam in the waist plane. Analytically, we derive at which polar angles intensity nulls can be located and define orders of the optical vortices born around these nulls. We also reveal which of the vortices contribute to the total TC of the superposition and which are compensated for by the opposite-sign vortices. If the LG beam has a TC of *m*, TC of the superposition is analytically shown to equal [*m*/2] or [*m*/2] + 1, where [] means an integer part of the fractional number. Thus, we show that the integer division of the TC by two can be done by superposing the LG beam with an off-axis Gaussian beam. Potential application areas are in optical computing machines and optical data transmission.

## 1. Introduction

In arithmetic, there are four basic operations: addition, subtraction, multiplication, and division. In computers, these operations are implemented in the processor, in the Assembler language, and are named ADD, SUB, MUL, and DIV, respectively. It is worth noting that when performed on some specific operands, these operations, on the one hand, are more often used in applications and, on the other hand, are easier to implement physically in computing devices. These operations include adding/subtracting a unit value, changing of sign, and multiplying/dividing by two. Therefore, these operations are implemented by special, faster processor routines and in the Assembler language they are known by separate commands: INC/DEC (add/subtract unit), NEG (change sign), and SHL/SHR (multiply/divide by 2). Although division is the most difficult arithmetic operation, the last two operations are implemented simply by shifting bits inside the byte, respectively, to the left or right.

In optical data processing and transmission, data are carried by structured light beams, with optical vortices showing promise. Research into the potential uses of OVs for data transmission is currently under way for both wireless [1,2] and fiber [3,4] communications. Because of its discreteness, one of the stable characteristics of optical vortices is the topological charge (TC) [5]. Even for scalar stochastic optical fields, the topological charge current has been shown to obey a conservation equation [6]. Unlike a bit in the computer, TC can theoretically have an infinite number of values. Therefore, possibilities have been investigated, on the one hand, for generating large-TC beams [7], and, on the other hand, for sorting the registered beam into a large number of channels with different TCs [8]. TC is often understood as the order of the optical vortex, but this is only correct for single-vortex beams. In the general case, TC was earlier defined by M.V. Berry as an integral over an infinite contour from the field phase derivative concerning the polar angle [5]. Methods for measuring TC based on Berry’s definition are currently being developed. For example, an optimal contour for the experimental measurement of TC by using a Shack–Hartmann sensor was studied in Ref. [9].

Along with data transmission, possibilities have been investigated for completing some operations with these data, including arithmetic ones. To perform addition/subtraction with a fixed operand, it is enough for an optical vortex to pass through a spiral phase plate (SPP). So, if an optical vortex with a TC of *m* passes through an *n*th order SPP, then the TC of the outgoing beam becomes *m* + *n*. The summation of TCs of two light beams is more difficult to implement. For example, the addition/subtraction of TCs is experimentally demonstrated in [10] in the process of four-wave mixing of optical vortices. The doubling of TC, as a partial case of TC summation, has been mentioned in the process of second harmonic generation [11]. A change of TC sign (TC inversion) can be implemented simply by reflecting the beam off the interface between the media (although the propagation direction changes in this case). TC inversion was also shown by using, for example, coil [12] and loop [13] resonators, as well as metasurfaces [14]. In our recent work [15,16], we showed that in the superposition of several coaxial Laguerre–Gaussian (LG) beams, the operation of choosing the maximum TC could be optically implemented, as the total TC of such a superposition exactly equalled the absolute value of the maximum TC of the constituent LG beams. To the best of our knowledge, no works dealing with dividing the TC of the beam by 2 have been published so far.

Meanwhile, many works have investigated the interaction of optical vortices. For example, the interference of LG modes with a plane wave and with LG modes carrying equal but opposite TC was experimentally studied in [17]. However, this work focused on the shape of the intensity pattern and on the number of petals rather than on the TC itself (although the number of petals allow for determining TC). The interaction of two LG beams with opposite TCs in a Sagnac interferometer was considered in [18], including in curved space–time, but the work was rather concerned with the interferometer sensitivity rather than with the TC. In [19], coherent Bessel vortex superposition with a linear charge increase was studied. That work mainly focused on the TC-independence of the beam radius and diffraction-free propagation. In [20], self-referenced interference of an optical vortex in a Mach–Zehnder interferometer was studied and the sign and magnitude of the TC were determined from the forks on the intensity pattern. In [21], elliptic perturbation of the optical vortex was investigated. That work focused on splitting the vortex into unit-strength vortices and their location.

In this work, we studied the TC of the superposition of an LG beam with zero radial (lower) index and an off-axis Gaussian beam in the waist plane. It was analytically shown that the intensity nulls in the transverse plane can reside only on a discrete set of polar angles. Orders of the vortices around these nulls were also obtained analytically and it was shown that TC carried by the superposed beam was defined only by vortices located in one transverse half-plane. It was proven that the TC of the superposition could have only two values: either TC of LG beam divided by 2 (integer part), or the next integer number. Potential application areas for this work are in optical computing machines where data are carried by vortex light beams and are encoded by the topological charges [22].

## 2. Topological Charge of Superposition of a Laguerre-Gaussian Beam with an Off-Axis Gaussian Beam: General Theory

Here, we studied the superposition of a zero-radial-index LG beam and an off-axis Gaussian beam with its center at the point (*a*, 0) (in the Cartesian coordinates) in the waist plane. The waist radii *w*_0_ of both beams were supposed to be equal. Without loss of generality, we also supposed that *a* > 0. The complex amplitude of such superposition reads as

(1) $$E\left(x,y\right)={\left(\frac{x+iy}{w_{0}}\right)}^{m}\exp\left(-\frac{x^{2}+y^{2}}{w_{0}^{2}}\right)+C\exp\left(-\frac{{\left(x-a\right)}^{2}+y^{2}}{w_{0}^{2}}\right),$$

where (*x*, *y*) are the Cartesian coordinates, *C* is the relative weight coefficient of the Gaussian beam, and *m* is the TC of the LG beam. We also suppose |*C*| ≠ 0, otherwise, the case is trivial. From now on, we use the normalized coordinates for brevity (*ξ* = *x*/*w*_0_, *η* = *y*/*w*_0_) and denote *α* = *a*/*w*_0_:

(2) $$E\left(\xi ,\eta \right)=e^{-\xi^{2}-\eta^{2}}\left[{\left(\xi +i\eta \right)}^{m}+Ce^{-\alpha^{2}+2\alpha \xi }\right]$$

To derive the TC of such a superposition, we need to find the optical vortices present in it. The coordinates (*ξ*_0_, *η*_0_) of nulls of the complex amplitude Equation (2) can be obtained from the following equation:

(3) $${\left(\xi_{0}+i\eta_{0}\right)}^{m}=\left|C\right|e^{im\Psi }e^{-\alpha^{2}+2\alpha \xi_{0}}$$

where the quantity *Ψ* = (arg *C* + π)/*m* characterizes (but is not equal to) the phase delay between the two interfering beams.

**Figure 1:**
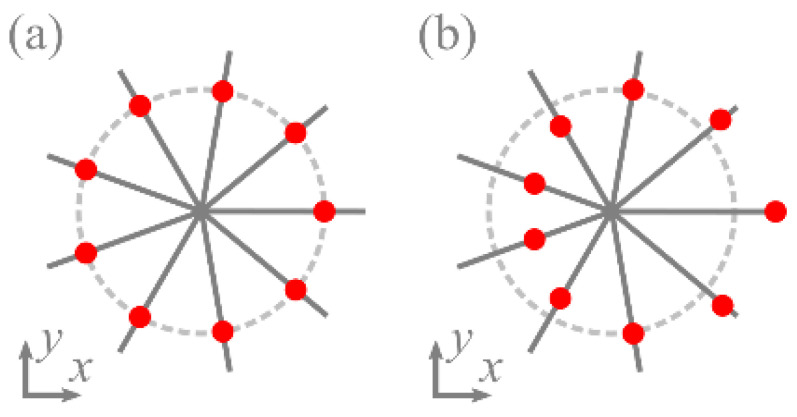
Locations of the intensity nulls on the polar angles in the superposition of a LG beam and an on-axis [21] (**a**) and off-axis (**b**) Gaussian beam.

From Equation (3), we obtain

(4) $$\xi_{0}+i\eta_{0}={\left|C\right|}^{1/m}e^{i\Psi }e^{-\alpha^{2}/m+2\alpha \xi_{0}/m}e^{2\pi ip/m}$$

with *p* = 0, …, *m* − 1. This equation is complex. Separating the real and imaginary parts yields

(5) $$\begin{array}{l} \xi_{0}={\left|C\right|}^{1/m}e^{-\alpha^{2}/m+2\alpha \xi_{0}/m}\cos\varphi_{p},\\ \eta_{0}={\left|C\right|}^{1/m}e^{-\alpha^{2}/m+2\alpha \xi_{0}/m}\sin\varphi_{p}, \end{array}$$

where

(6) $$\varphi_{p}=\Psi +\frac{2\pi }{m}p$$

are the polar angles where the intensity nulls can reside. Note that these polar angles take discrete values, as is the case with the coaxial superposition of LG and the Gaussian beams considered in [21] (Figure 1a). The difference from [21] is that for each angle *φ_p_* the intensity nulls are at a different distance from the center (Figure 1b).

In the second equation in (5), *η*_0_ is expressed explicitly. In the first equation, to simplify the argument of the exponent, we replace *ξ*_0_ by *ms*/(2*α*), where *s* is a scaled horizontal Cartesian coordinate of the sought-for intensity null (if this null resides in a certain point (*x*_0_, *y*_0_), then *s* = 2α*x*_0_/(*mw*_0_)). Thus, the first equation in (5) takes the following form:

(7) $$s=Ae^{s}$$

where *A* = (*eV*)^−1^cos *φ_p_* and

(8) $$V=\frac{m}{2e\alpha {\left|C\right|}^{1/m}}e^{\alpha^{2}/m}$$

is a “vorticity” parameter, because, as we show below, it affects the number of vortices in the directions defined by the polar angles *φ_p_*, given by Equation (6) (there can be zero, one, or two vortices depending on what value is greater: *V* or cos *φ_p_*).

**Figure 2:**
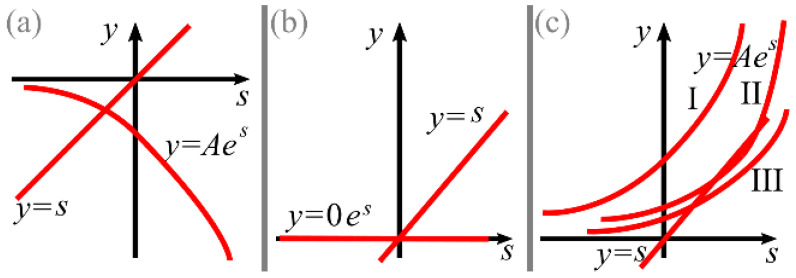
Possible cases when solving an equation *s* = *Ae^s^*: *A* < 0 (**a**), *A* = 0 (**b**), *A* > 0 (**c**).

Generally, such an equation cannot be solved analytically. However, it is obvious (Figure 2) that, depending on the coefficient *A* from Equation (7), it can have 0, 1, or 2 roots.

If *A* < 0 [Figure 2a], there is only one negative root *s* < 0.

If *A* = 0 [Figure 2b], there is only one root *s* = 0.

If *A* > 0 [Figure 2c], there can be 0, 1, or 2 roots. The case with one root is easy to guess as the equation *e^s^* = *es* has the only root *s* = 1. So, there are no roots if *A* > 1/*e* (curve I in Figure 2c), there is one root if *A* = 1/*e* (curve II in Figure 2c), and there are two roots if 0 < *A* < 1/*e* (curve III in Figure 2c), one of which is in the interval (0, 1), and the other are in (1, ∞).

So, we obtain from Equation (5) the number of intensity nulls residing at each polar angle *φ_p_*.

If cos *φ_p_* < 0, there is one intensity null (*ξ*_0_ < 0) in the direction *φ_p_*.

If cos *φ_p_* = 0, the intensity null is on the vertical axis (*ξ*_0_ = 0).

If 0 < cos *φ_p_* < *V*, there are two intensity nulls, so that 0 < *ξ*_0_ < *m*/(2*α*) for one null and *ξ*_0_ > *m*/(2*α*) for the other.

If cos *φ_p_* = *V*, there is one null with *ξ*_0_ = *m*/(2*α*).

If cos *φ_p_* > *V*, there are no intensity nulls in this direction.

Below, we study if there are optical vortices around these intensity nulls (*ξ*_0_, *η*_0_), and which order these vortices have. To do this, we consider a point in the vicinity of the intensity null, i.e., a point (*ξ*, *η*) = (*ξ*_0_ + *r* cos *φ*, *η*_0_ + *r* sin *φ*) with *r* being a small distance:

(9) $$E\left(\xi ,\eta \right)=e^{-\xi^{2}-\eta^{2}}\left[{\left(\xi_{0}+i\eta_{0}+re^{i\varphi }\right)}^{m}+Ce^{-\alpha^{2}+2\alpha \xi_{0}+2\alpha r\cos\varphi }\right]$$

Gaussian envelope exp(–*ξ*^2^ − *η*^2^) does not affect the phase distribution. In the square brackets, doing the binomial expansion of the first term and series expansion of the exponent exp(2*αr*cos *φ*) in the second term, after neglecting the terms *r*^2^, *r*^3^, etc., we obtain:

(10) $$E\left(\xi ,\eta \right)=e^{-\xi^{2}-\eta^{2}}[{\left(\xi_{0}+i\eta_{0}\right)}^{m}+m{\left(\xi_{0}+i\eta_{0}\right)}^{m-1}re^{i\varphi }+Ce^{-\alpha^{2}+2\alpha \xi_{0}}\left(1+2\alpha r\cos\varphi \right)].$$

As the amplitude is zero at the point (*ξ*_0_, *η*_0_), then

(11) $${\left(\xi_{0}+i\eta_{0}\right)}^{m}+Ce^{-\alpha^{2}+2\alpha \xi_{0}}=0$$

and so Equation (10) reads as

(12) $$E\left(\xi ,\eta \right)=re^{-\xi^{2}-\eta^{2}}\left\{m{\left(\xi_{0}+i\eta_{0}\right)}^{m-1}e^{i\varphi }+2Ce^{-\alpha^{2}+2\alpha \xi_{0}}\alpha \cos\varphi \right\}.$$

Using Equation (11) again, we replace in Equation (12) $Ce^{-\alpha^{2}+2\alpha \xi_{0}}$ by $-{\left(\xi_{0}+i\eta_{0}\right)}^{m}$:

(13) $$E\left(\xi ,\eta \right)=re^{-\xi^{2}-\eta^{2}}{\left(\xi_{0}+i\eta_{0}\right)}^{m-1}\left[me^{i\varphi }-2\left(\xi_{0}+i\eta_{0}\right)\alpha \cos\varphi \right].$$

The vortex order (local topological charge) at the point (*ξ*_0_, *η*_0_) is defined by the expression in the square brackets, and (see Appendix A) it equals

(14) $$TC=\mathrm{sgn}\left\{m-2\alpha \xi_{0}\right\}$$

Thus, the orders of the optical vortices in the superposition (1) are given in Table 1.

**Figure 3:**
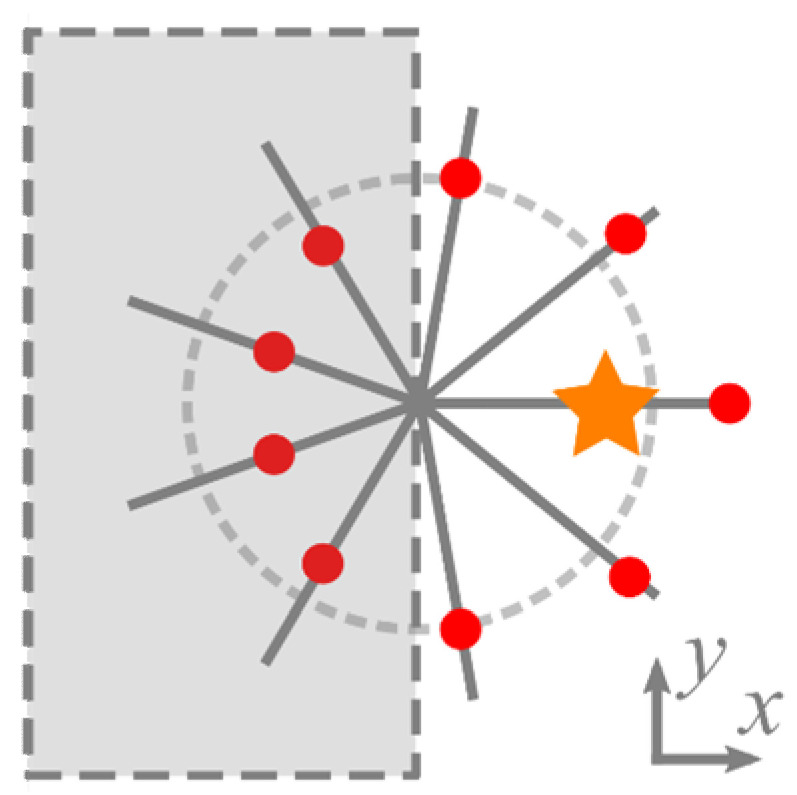
Contribution of the intensity nulls of the superposition (1) into the topological charge. Red circles are the intensity nulls, an orange star is the off-axis Gaussian beam, and the grey half-plane is the one where the intensity nulls contribute to TC (other nulls do not contribute).

Table 1 suggests that the total TC of the whole superposition constitutes only those intensity nulls that reside on the polar angles with π/2 ≤ *φ_p_* ≤ 3π/2, i.e., in one transverse half-plane (Figure 3). For other directions *φ_p_*, either there are no intensity nulls, or there is one intensity null without a vortex around it, or there are two nulls with optical vortices of opposite orders so that they do not affect the total TC.

Therefore, if the LG beam carries a TC of *m*, then, after interfering with a Gaussian beam, the TC becomes either [*m*/2] or [*m*/2 + 1] ([] is the integer part of a fractional number). This means that the off-axis Gaussian beam can be used to implement the operation of the integer division by 2 of the TC of the LG beam (optical analogue of shifting all bits in a byte to the right, Assembler command SHR).

We note that the angles *φ_p_* are independent of the Gaussian beam amplitude |*C*|. Thus, TC depends only on the phase delay arg *C* between the beams. It seems strange enough, as in the absence of the Gaussian beam (|*C*| → 0), there is no way to change the TC of the LG beam. However, the apparent collision is explainable. According to Table 1, when |*C*| → 0, there are two intensity nulls for each angle *φ_p_* with cos *φ_p_* > 0. However, the second null goes to infinity at |*C*| → 0 and disappears completely at |*C*| = 0, making TC equal to *m* instead of [*m*/2] or [*m*/2] + 1.

Further on, we consider particular simple cases of superposition (*m* = 1, 2, 4).

## 3. Topological Charge of Superposition of a LG Beam and an Off-Axis Gaussian Beam: Particular Cases

If *m* = 1, the intensity nulls can reside only at the polar angle *φ*_0_ = *Ψ*. If the absolute phase delay between the beams |arg *C*| is less than π/2, then the TC of the LG beam remains equal to 1, independently of the Gaussian beam power. Otherwise, TC becomes equal to zero, although the beam can have up to two intensity nulls, depending on the sign of the quantity cos *φ_p_* − exp(*α*^2^)/(2*eα*|*C*|^1/*m*^). For example, for *α* = 1 (shift of the Gaussian beam equals its waist radius), arg *C* = π (antiphase superposition), a number of intensity nulls depend on the sign of |*C*| − 1/2. If *C* = −1, there are no intensity nulls (bottom row in Table 1). If *C* = −1/2 (next-to-last row in Table 1), there is an intensity null, but without the vortex (phase on a contour around the null increases and decreases without 2π jumps). If *C* = −1/10, the Gaussian beam affects the intensity distribution of the LG beam weakly, but two intensity nulls simple with optical vortices of the opposite sign.

**Figure 4:**
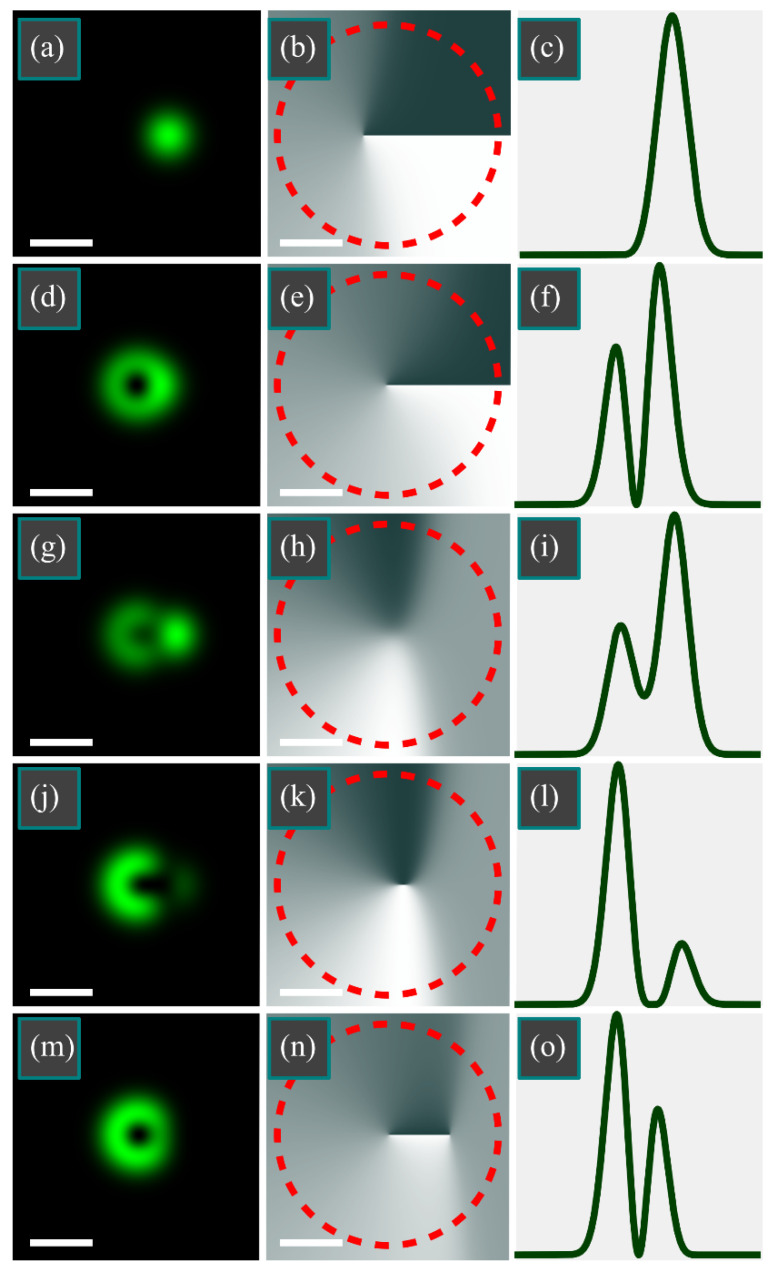
Distributions of intensity (**a**,**d**,**g**,**j**,**m**) and phase (**b**,**e**,**h**,**k**,**n**), as well as normalized-to-maximum horizontal intensity cross-sections (**c**,**f**,**i**,**l**,**o**) of the superposition of the LG beam with an off-axis Gaussian beam for the following parameters: wavelength *λ* = 532 nm; waist radius *w*_0_ = 0.5 mm; azimuthal index of LG beam *m* = 1; transverse shift of the Gaussian beam *a* = *w*_0_; Gaussian beam weight coefficient *C* = 10 (**a**–**c**), *C* = 0.1 (**d**–**f**), *C* = −1 (**g**–**i**), *C* = −0.5 (**j**–**l**), and *C* = −0.1 (**m**–**o**); calculation domain −*R* ≤ *x*, *y* ≤ *R* (*R* = 2 mm); and number of points *N* = 1024. TC was computed along the dashed circle (**b**,**e**,**h**,**k**,**n**). The white scale mark in the left bottom area of each Figure denotes 1 mm.

Figure 4 shows the intensity and phase distributions of the superposition of the LG beam and an off-axis Gaussian beam for the following parameters: wavelength *λ* = 532 nm, waist radius *w*_0_ = 0.5 mm; azimuthal index of LG beam *m* = 1; a transverse shift of the Gaussian beam *a* = *w*_0_; Gaussian beam weight coefficients of *C* = 10 (Figure 4a–c), *C* = 0.1 (Figure 4d–f), *C* = −1 (Figure 4g–i), *C* = −0.5 (Figure 4j–l), and *C* = −0.1 (Figure 4m–o); calculation domain –*R* ≤ *x*, *y* ≤ *R* (*R* = 2 mm); and number of points *N* = 1024 (along both coordinate axes).

In Figure 4a–c, the Gaussian beam is much brighter compared with the LG beam, in contrast with Figure 4d–f, but the TC of the LG beam in both cases is unaffected by the Gaussian beam and remains equal to 1. If the beams have a phase difference of π (Figure 4g–o), then, regardless of their amplitudes, the TC is zero: either there are no intensity nulls (Figure 4g–i), or there is one intensity null (*x* = *w*_0_/2 = 0.25 mm) without an optical vortex (Figure 4j–l), or there are two opposite-sign vortices (Figure 4m–o).

If *m* = 2, the only possibility for TC to remain equal to 2 is when there is no phase delay between the beams (*Ψ* = π/2). If there is even a small phase difference, one of the angles *φ*_0_, *φ*_1_ is in the range (−π/2, π/2) and thus TC becomes equal to 1. The number of intensity nulls can also be different, e.g., at *α* = 1 and arg *C* = π, it depends on the sign of |*C*| − 1/*e*.

**Figure 5:**
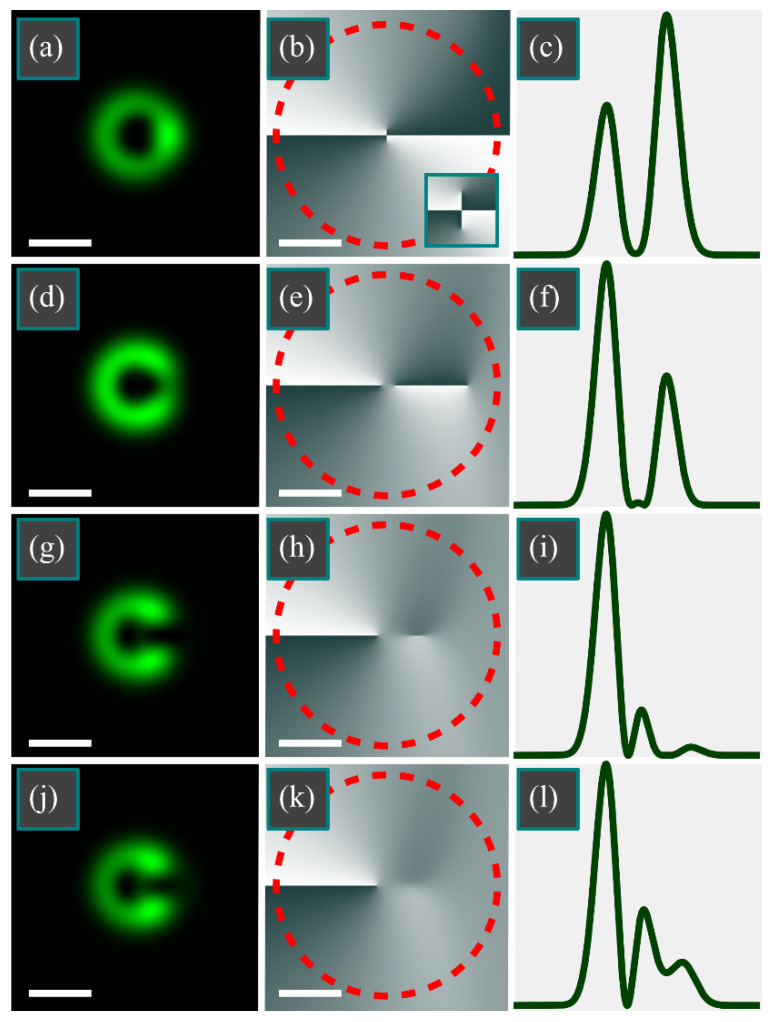
Distributions of intensity (**a**,**d**,**g**,**j**) and phase (**b**,**e**,**h**,**k**), as well as normalized-to-maximum horizontal intensity cross-sections (**c**,**f**,**i**,**l**) of the superposition of the LG beam with an off-axis Gaussian beam for the following parameters: wavelength *λ* = 532 nm; waist radius *w*_0_ = 0.5 mm; azimuthal index of LG beam *m* = 2; the transverse shift of the Gaussian beam *a* = *w*_0_; Gaussian beam weight coefficient *C* = 0.1 (**a**–**c**), *C* = −0.1 (**d**–**f**), *C* = −1/*e* (**g**–**i**), and *C* = −0.5 (**j**–**l**); calculation domain −*R* ≤ *x*, *y* ≤ *R* (*R* = 2 mm); and number of points *N* = 1024. TC was computed along the dashed circle (**b**,**e**,**h**,**k**). The white scale mark in the left bottom area of each Figure denotes 1 mm. The inset (**b**) shows the central area with 3× zoom.

Figure 5 depicts the intensity and phase distributions of the superposition of the LG beam with an off-axis Gaussian beam for the following parameters: wavelength *λ* = 532 nm; waist radius *w*_0_ = 0.5 mm; azimuthal index of LG beam *m* = 2; a transverse shift of the Gaussian beam *a* = *w*_0_; Gaussian beam weight coefficient *C* = 0.1 (Figure 5a–c), *C* = −0.1 (Figure 5d–f), *C* = −1/*e* (Figure 5g–i), and *C* = −0.5 (Figure 5j–l); calculation domain −*R* ≤ *x*, *y* ≤ *R* (*R* = 2 mm); number of points *N* = 1024.

According to Figure 5a–c, the Gaussian beam does not change the TC of the LG beam, which remains equal to 2. If the beams have a phase difference of π (Figure 5d–l), then, regardless of their amplitudes, TC equals 1: either there are three intensity nulls, but two of them have opposite-sign vortices (Figure 5d–f), or there are two intensity nulls, but one of them is without an optical vortex (*x* = *w*_0_ = 0.5 mm) (Figure 5g–i), or there is only one vortex (Figure 5j–l).

Similar to the cases considered above, if *m* = 4, then the intensity nulls can reside only at the polar angles *φ_p_* = *Ψ* + π*p*/2 (*p* = 0, 1, 2, 3).

If there is no phase delay between the beams (arg *C* = 0 and *Ψ* = π/4), there are two angles *φ_p_*, such that cos *φ_p_* ≤ 0 and thus the TC of the whole superposition equals 2. TC can be made equal to 3 if the beams have a phase delay of π (arg *C* = π and *Ψ* = π/2), as in this case, there are three angles *φ_p_*, such that cos *φ_p_* ≤ 0: *φ*_0_ = π/2, *φ*_1_ = π, *φ*_2_ = 3π/2.

**Figure 6:**
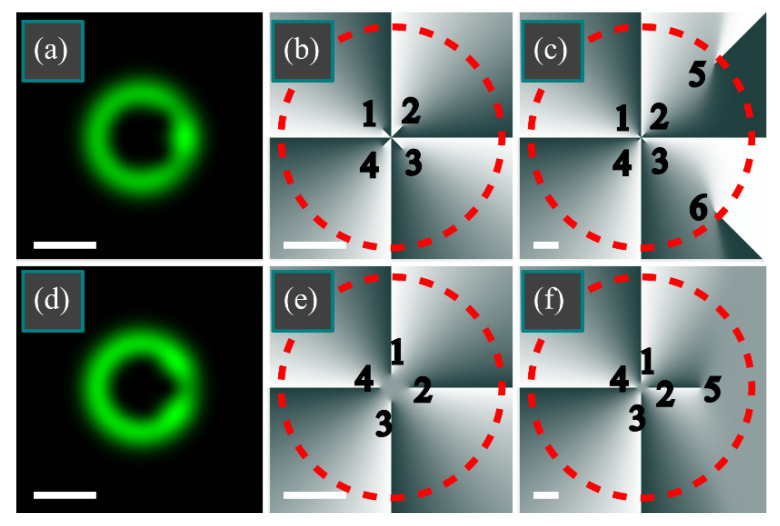
Distributions of intensity (**a**,**d**) and phase (**b**,**c**,**e**,**f**) of the superposition of the LG beam with an off-axis Gaussian beam for the following parameters: wavelength *λ* = 532 nm, waist radius *w*_0_ = 0.5 mm, azimuthal index of LG beam *m* = 4, a transverse shift of the Gaussian beam *a* = *w*_0_, Gaussian beam weight coefficient *C* = 0.1 (**a**–**c**) and *C* = −0.1 (**d**–**f**), calculation domain −*R* ≤ *x*, *y* ≤ *R* with *R* = 2 mm (**a**,**b**,**d**,**e**) and *R* = 5 mm (**c**,**f**), and number of points *N* = 1024. TC was computed along the dashed circle (**b**,**c**,**e**,**f**). The white scale mark in the left bottom area of each Figure denotes 1 mm. Black digits in phase distributions (**b**,**c**,**e**,**f**) denote vortices.

Figure 6 illustrates the intensity and phase distributions of such superpositions for the following parameters: wavelength *λ* = 532 nm, waist radius *w*_0_ = 0.5 mm, azimuthal index of LG beam *m* = 4, a transverse shift of the Gaussian beam *a* = *w*_0_, Gaussian beam weight coefficient *C* = 0.1 (Figure 6a–c) and *C* = −0.1 (Figure 6d-f), calculation domain −*R* ≤ *x*, *y* ≤ *R* with *R* = 2 mm in Figure 6a,b,d,e and *R* = 5 mm in Figure 6c,f, and number of points *N* = 1024.

As seen in Figure 6a,d, the intensity ring is weakly distorted by a Gaussian beam and in Figure 6b,e, TC can be estimated to remain equal to 4. The calculation along the dashed circles in Figure 6b,e also yields 4. However, for a wider domain, *R* = 5 mm, it is seen that there are six vortices in Figure 6c: four vortices that carry TC +1 (vortices 1–4) and two vortices that carry TC −1 (vortices 5,6). Therefore, the TC of the whole beam becomes equal to 2. Similarly, there are five vortices in Figure 6f: four vortices with TC + 1 (vortices 1–4) and one vortex with TC −1 (vortex 5). Thus, the TC of the whole beam is 3. Figure 6 confirms the theory, i.e., that the TC is contributed by the vortices residing in the left side of the transverse plane. For instance, in Figure 6c, the TC is contributed by vortices 1 and 4, whereas the vortices 2 and 3 are compensated by the vortices 5 and 6, respectively. Similarly, in Figure 6f, TC is contributed by vortices 1, 3, and 4, whereas vortex 2 is compensated by vortex 5.

## 4. Conclusions

Thus, we have proven theoretically and confirmed numerically that the TC of a single-ringed (zero-radial-index) LG beam can be optically divided by 2 through interference with an off-axis Gaussian beam. We considered only a simple case when the waist radii of the perturbed LG beam and of the off-axis Gaussian beam were the same. The proof is based on determining the positions of the intensity nulls and the orders of the vortices around them (local topological charges). It has been shown analytically that only the intensity nulls located in the half-plane perpendicular to the propagation direction contribute to the TC of the superposition. If the TC of the LG beam is even and equal *m*, then after perturbing by an off-axis Gaussian beam, the TC of a superposition is equal to *m*/2 + 1 (if the phase delay between the beams is arg *C* = π*m*/2 ± π) or *m*/2 (for other phase delays). If the TC *m* of the LG beam is odd, then the TC of a superposition is equal to (*m* + 1)/2 (if the phase delay arg *C* between the beams is in the interval [π*m*/2 − π, π*m*/2]) or (*m* − 1)/2 (for the phase delays in the interval (π*m*/2, π*m*/2 + π)). The amplitude |*C*| of the perturbing Gaussian beam does not affect the TC of the superposition, but affects the position of the optical vortices and, thus, the radius of the correct TC computation. The numerical simulation confirmed our theory and demonstrated in simple cases the integer division of TC by 2. In the numerical simulation, it was the most difficult to calculate the amplitude and phase in the regions of a very low intensity (in the math software we used, the intensity in those regions was assumed to be zero); therefore, we were unable to demonstrate the division by 2 of high TC values. In the real experiment, there is the same measurement problem, which, in addition, becomes more complicated due to noises. However, we derived the TC value only in the initial plane, whereas in other planes, this derivation would be more cumbersome, if it was even possible. Nevertheless, upon propagation, optical vortices can move to infinity or come back from it [23]. Thus, the optical vortices from low-intensity areas in one plane can become measurable in other transverse planes, for instance in the focal plane, or at a certain specific distance in space, but we did not investigate determining such planes in this work. In the future, we are going to extend this study, as it is unknown yet how TC changes if the LG beam with a zero radial index is replaced by a LG beam of an arbitrary radial order, or if the Gaussian beam is replaced by another field. The behavior of the optical vortices in the considered superposition upon free-space propagation has also remained unstudied. The potential application area is in optical computing machines where data are carried by vortex light beams and are encoded by the topological charges [24,25].

## Figures and Tables

**Table 1:** Intensity nulls and their orders (local topological charges).

*φ_p_*	ξ_0_	TC
cos *φ_p_* < 0	ξ_0_ < 0	+1
cos *φ_p_* = 0	ξ_0_ = 0	+1
0 < cos *φ_p_* < *V*	0 < ξ_0_ < *m*/(2α)	+1
	ξ_0_ > *m*/(2α)	–1
cos *φ_p_* = *V*	ξ_0_ = *m*/(2α)	0
cos *φ_p_* > *V*	-	

## Data Availability

Not applicable.

## Appendix A

We suppose that (*r*, *φ*) are the polar coordinates in the vicinity of a null of some function, and this function is approximately equal to

f(φ)=acosφ+bsinφ

with *a* and *b* being some complex coefficients.

Along some contour around this null of the function, the phase changes the following number of 2π (local topological charge):

TC=12πIm∫02π∂f/∂φfdφ=12πIm∫02πbcosφ−asinφacosφ+bsinφdφ

This integral can be represented as an integral over a unit-radius circle in the complex plane (*z* = *e^iφ^*):

TC=12πIm∫02πb(z+z−1)+ia(z−z−1)a(z+z−1)−ib(z−z−1)dziz

Multiplying the numerator and denominator by *z*, we obtain the integral

TC=12πIm∫02π(a−ib)z2−(a+ib)(a−ib)z2+(a+ib)dzz

which depends on the number of poles inside the unit-radius circle. There can be either one pole (*z* = 0), or this pole and two additional poles, such that

$$z^{2}=-\frac{a+ib}{a-ib}$$

The second case occurs if |*a* + *ib*| < |*a* − *ib*|, which is similar to the condition

Ima⋅Reb<Rea⋅Imb

Thus, if Im *a*⋅Re *b* > Re *a*⋅Im *b*, there is only pole (*z* = 0) and the integral is easily obtained:

TC=12πIm{2πi−(a+ib)(a+ib)}=−1

If Im *a*⋅Re *b* < Re *a*⋅Im *b*, there are two poles, *z*_1_ and *z*_2_ (*z*_1_ + *z*_2_ = 0 and *z*_1_⋅*z*_2_ = (*a* + *ib*)/(*a* − *ib*)). So,

TC=12πIm∫02π(z−iz1)(z−iz2)(z−z1)(z−z2)dzz

Adding the values given by all three poles, we obtain

TC=−1+12πIm{2πi(z1−iz1)(z1−iz2)(z1−z2)z1+2πi(z2−iz1)(z2−iz2)(z2−z1)z2}.

Simplification yields

TC=−1+Im{i(1−i)z2−z1[−(z1−iz2)+(z2−iz1)]} =−1+Im{i(1−i)(1+i)}=1.

Finally, if Ima⋅Reb=Rea⋅Imb, then *b*/*a* is a real number and f(φ)=a(cosφ+(b/a)sinφ), i.e., the amplitude changes around the null, while the phase does not change. Thus, the local TC is zero.

To summarize the three considered cases, we conclude that the local topological charge in the vicinity of the null of a function f(φ)=acosφ+bsinφ is equal to

TC=sgn{Rea⋅Imb−Ima⋅Reb}

with sgn being the sign function (sgn ξ = 1 at ξ > 0, sgn ξ = −1 and ξ < 0, and sgn 0 = 0).
